# Oncological microdissection testicular sperm extraction (Onco‐microTESE) outcomes for fertility preservation of patients with testicular cancer with azoospermia or severe oligoasthenoteratozoospermia

**DOI:** 10.1111/bju.16553

**Published:** 2024-11-16

**Authors:** Jack B. Fanshawe, Thomas Hughes, Karen Briggs, Raveen Sandher, Yacoub Khalaf, Tet Yap, Julia Kopeika, Majid Shabbir

**Affiliations:** ^1^ Department of Urology Guy's Hospital London UK; ^2^ The Assisted Conception Unit Guy's Hospital London UK; ^3^ King's College London London UK

**Keywords:** azoospermia, fertility preservation, oligoasthenoteratozoospermia, surgical sperm retrieval, testicular cancer

## Abstract

**Objective:**

To determine the success rate of oncological microdissection testicular sperm extraction (onco‐microTESE) in patients with testicular cancer (TC) with azoospermia and severe oligoasthenoteratozoospermia (OAT; <1 million/mL sperm) and to explore any factors that may predict success.

**Patients and Methods:**

Case series of outcomes from all consecutive patients (42 testes in 38 patients) that presented or were referred to a single specialist tertiary referral centre for fertility management in the context of TC with severe OAT or azoospermia between August 2015 and August 2022. Biochemical, radiological, and histological parameters were collected for all patients. All patients underwent onco‐microTESE (simultaneous radical inguinal orchidectomy with *ex vivo* microTESE of the affected testis). Those with unsuccessful surgical sperm retrieval (SSR) from the affected testis underwent contemporaneous contralateral microTESE, if no contraindication was present. The primary outcome was successful SSR from the affected testicle sufficient for assisted reproductive techniques. Secondary outcomes included contralateral microTESE success, the time from referral to procedure, and the total successful fertility preservation rate.

**Results:**

Initial onco‐microTESE was successful in 19 of 31 patients (61%) with azoospermia. Contralateral microTESE was successful in a further two of eight patients with azoospermia with failed onco‐microTESE. Overall, 22/31 patients with azoospermia (71%) had successful fertility preservation in this series. In addition, six of seven patients with severe OAT had further sperm harvested by onco‐microTESE to maximise their fertility preservation. All surgery was performed within median (interquartile range) of 7 (5–13) days from presentation.

**Conclusions:**

Onco‐microTESE represents an effective method of fertility preservation for sub‐fertile patients with TC without delaying oncological treatment. Knowledge of the fertility status at first presentation is essential to allow for such additional options for optimal fertility preservation in TC.

**Abbreviations**
AFP: alpha fetoprotein
ART: assisted reproductive technology
EAU: European Association of Urology
GCNIS: germ cell neoplasia *in situ*
ICSI: intracytoplasmic sperm injection
IQR: interquartile range
LDH: lactate dehydrogenase
OAT: oligoasthenoteratozoospermia
onco: oncological
PTTO: percentage testis tumour occupation
RPLND: retroperitoneal lymph node dissection
SSR: surgical sperm retrieval
(micro)TESE: (microdissection) testicular sperm extraction
TC: testicular cancer
US: ultrasonography

## Introduction

Testicular cancers (TCs) are most common in men aged <40 years [1] coinciding with their prime fertile years. In all, 45% of men diagnosed with TC are sub‐fertile on presentation [2], and up to 6–24% are azoospermic [2, 3]. Understanding the fertility preservation options for this significant cohort of patients is of key importance. As overall sperm counts of men fall worldwide, the sub‐fertile patient with TC may become an increasingly common management dilemma for urologists and oncologists [4].

Fertility concerns amongst patients with TC can lead to significant additional anxiety and distress [5]. In all, 76% of childless male patients with cancer desire to have a family in the future [6]. Given the excellent improvements in long‐term survival in TC [7], concerns about the impact of the tumour and its treatment on future fertility are high [8]. Offering effective fertility preservation may improve anxiety and long‐term quality of life [8].

Testicular cancer and impaired semen quality share genetic, environmental and lifestyle risk factors and may represent two manifestations of the testicular dysgenesis syndrome [7]. The TC itself may inhibit spermatogenesis at both the hormonal level, via disruption of the hypothalamic–pituitary–gonadal axis, and the cellular level through oxidative stress and DNA fragmentation [9, 10, 11, 12]. Orchidectomy compounds fertility further by reducing sperm concentration by ~25%, and whilst levels can return to baseline in the subsequent year [13], up to 9% may become azoospermic [14]. The gonadotoxic effects of adjuvant therapies, such as chemotherapy and radiotherapy, are well documented, whilst retroperitoneal lymph node dissection (RPLND) may lead to ejaculatory dysfunction [13, 15, 16].

Given the significant potential impact of treatment on fertility potential, the European Association of Urology (EAU) guidelines recommend fertility preservation prior to any intervention [17]. Cryopreservation is the most efficient and cost‐effective method of fertility preservation in those able to produce a sample containing sperm. In patients with azoospermia, surgical sperm retrieval (SSR) techniques have vastly improved chances of fertility. For those with non‐obstructive azoospermia due to testicular dysfunction, microdissection testicular sperm extraction (microTESE) is currently recommended by the EAU for SSR. MicroTESE has been adapted for patients with azoospermia undergoing orchidectomy for TC. An ‘Onco‐microTESE’ involves performing a radical inguinal orchidectomy combined with a contemporaneous ex vivo microTESE of the removed testis. This technique allows the surgeon to identify suitable seminiferous tubules away from the tumour where sperm extraction success is enhanced [18].

This study aimed to ascertain the success rate of onco‐microTESE procedures and investigate what clinical factors, if any, may influence or predict the chance of success.

## Patients and Methods

### Study Design

Retrospective review of a prospectively collated database of all patients that underwent onco‐microTESE at Guy's Hospital, London, UK from the service's inception in August 2015 to August 2022.

### Patient Identification and Data Collection

All patients aged ≥16 years referred, or presenting, with testicular masses suspicious of a malignancy and azoospermia or poor‐quality ejaculated sperm (i.e., very severe oligoasthenoteratozoospermia [OAT] with sperm counts of <1 million/mL with poor motility and reduced normal forms) who underwent onco‐microTESE after review and discussion in the multidisciplinary team meeting. All patients provided a total of two samples for semen analysis. All patients presenting with severe OAT were offered cryopreservation on multiple occasions prior to surgery.

If no sperm were found in the affected testicle, a contralateral microTESE was performed from the unaffected testicle at the same sitting, and data on the success of this procedure was collated and recorded separately. No patients received hormone stimulation prior to onco‐microTESE.

Tumour markers (beta‐hCG, lactate dehydrogenase [LDH], alpha‐fetoprotein [AFP]) and hormone profiles (FSH, LH and testosterone) were measured before orchidectomy. All patients had semen analysis at first presentation and results were reported according to the WHO fifth edition reference ranges [19]. Testis and tumour volumes were calculated from ultrasonography (US) and pathological specimens separately, using the volume of an ellipsoid object formula (*V* = 4/3π*abc*). Percentage testis tumour occupation (PTTO) was defined as tumour volume/testis volume.

The US scans were reported by experienced uro‐radiologists and ultrasonographers at first presentation to Guy's Hospital. All histopathology reports were compiled by experienced histopathologists at Guy's Hospital.

### Outcome Measures

The primary outcome measure was the successful extraction of sperm intraoperatively from the affected testicle; this was defined as spermatozoa being retrieved of sufficient quality and quantity suitable for freezing and future use in assisted reproductive technologies (ARTs). Secondary outcome measures included the number of vials successfully stored, the need for, and success of, any contralateral microTESE if performed, the time to surgery from referral, and the overall fertility preservation rate for all patients referred to the service.

Outcomes in patients with azoospermia were assessed separately to those with very severe OAT. Within these two cohorts a comparison of clinical variables was made between participants that underwent successful SSR and unsuccessful SSR.

### Surgical Technique

An Onco‐microTESE was defined as an ipsilateral *ex vivo* microTESE at the time of radical orchidectomy for TC. All onco‐microTESE cases were performed by one of three high‐volume Consultant Uro‐andrologists (T.Y., R.S., M.S.) who were fellowship trained and practising in one of the UK's largest tertiary referral centre for male fertility services with a referral base population of >8 million, where an average of 1–2 microTESEs are performed every week.

Radical orchidectomy was performed in the standard way, with an inguinal incision and soft clamping of the cord before mobilisation of the testes out of the scrotum. After ligation and transection of the cord, the affected testicle was transferred to a prepared bench for *ex vivo* examination using a Zeiss operating microscope while the inguinal wound was closed. The tunica albuginea was opened away from the palpable tumour, and the seminiferous tubules of the bivalved testis were examined at high power (up to ×15–×20). Engorged opaque tubules most likely to represent areas of focal spermatogenesis were removed (2–10 mg samples) and immediately examined simultaneously in theatre by the attending experienced embryologist using a light microscope, with further analysis using an inverted microscope in the laboratory as required. The surgically retrieved testicular biopsy was placed into MOPS+ media and mechanical dissection of tubules was undertaken to facilitate the release of sperm. After thorough dissection, the supernatant was transferred to another vial and a drop of supernatant was examined again under the microscope. If sperm of sufficient quantity and quality was identified for one intracytoplasmic sperm injection (ICSI) as determined by an experienced embryologist, freezing could be performed. If the concentration of visible sperm was below this threshold, further centrifugation at 300g for 10 min was undertaken. If no sperm was found on the ipsilateral side, a contralateral microTESE was performed at the same sitting via a separate scrotal incision. Success from the contralateral testis was reported separately to the successful onco‐microTESE from the affected testicle. Successfully extracted samples were subsequently taken to the embryology laboratory, where they were centrifuged and further analysed using a high‐power inverted microscope.

### Ethical Approval

Due to its design, ethical approval was not required for this study, and it was prospectively registered as a service evaluation within the hospital trust.

### Statistical Analysis

Statistical analysis was performed using the Statistical Package for the Social Sciences (SPSS®), version 28 (IBM Corp., Armonk, NY, USA).

Results are presented as mean (sd) or median and interquartile range (IQR) for parametric and non‐parametric variables respectively.

## Results

### Baseline Characteristics and Outcomes

Overall, 42 testicular units underwent onco‐microTESE from 38 patients (Table 1). In all, 31 patients (35 testes) were azoospermic and seven patients (seven testes) had severe OAT (poor quality sperm with counts <1 million/mL). Four of the patients with azoospermia had bilateral synchronous tumours, 10 patients with azoospermia presented with tumour in a solitary functioning testis. The median (IQR) age of the cohort was 34 (29–36) years. The tumour staging was pT1 in 27 testicular units, pT2 in four and not available in four due to tumour regression. Leydig cell tumours were reported in the remaining seven. The histopathological classification was seminoma (23), Leydig cell neoplasm (seven) mixed germ cell (six), burned out (four), non‐seminomatous germ cell (two). FSH was double the upper limit of normal in both the successful and unsuccessful groups (median 25 and 26 IU/mL, respectively). No patients had immediate postoperative complications. Patients that required bilateral orchidectomy were treated with testosterone replacement therapy as per local guidelines.

**Table 1:** Summary of baseline characteristics.

Characteristic	Value
Degree of infertility, *n* Azoospermia Severe OAT	31 7
Tumour staging, *n* pT1 pT2 No stage (tumour regression, Leydig cell)	27 4 11
Histological classification, *n* Seminoma Leydig cell tumour Mixed germ cell Burned out tumour Non‐seminomatous germ cell	23 7 6 4 2
Age, years, median (IQR)	34 (29–36)
AFP (normal range: 0–5.8 kU/mL), kU/mL median (IQR)	2.0 (1.6–6.4)
Beta‐hCG (normal range: 0–2 mIU/mL), mIU/mL, median (IQR)	<1 (0.0–2.0)
LDH (normal range: 140–225 U/L), U/L, median (IQR)	187 (160–218)
Total testosterone (normal range: 9.9–30 nmol/L), nmol/L, median (IQR)	11.0 (7.0–17.5)
FSH (normal range: 1–12.4 IU/L), IU/L, median (IQR)	26.0 (9.9–33.1)
LH (normal range: 1–8.6 IU/L), IU/L, median (IQR)	10.8 (4.9–16.4)
Testes volume on US, mL, median (IQR)	8.3 (6.2–13.3)
Tumour volume on US, mL, median (IQR)	1.7 (0.5–6.3)
PTTO on US, %, median (IQR)	21 (8–36)
Maximum tumour length on US, cm, median (IQR)	1.5 (1.1–2.3)
Time from presentation to operation, days, median (IQR)	7 (5–13)

The median (IQR) time from presentation or referral received at Guy's hospital to surgery was 7 (5–13) days. Of patients followed up at Guy's hospital (*n* = 22), the median (IQR) follow‐up was 32 (16–38) months. In all, 16 patients were referred back to their local hospital or are yet to be seen due to their operation date. Six patients received adjuvant chemotherapy after orchidectomy. One underwent RPLND and adjuvant chemotherapy, and a further patient underwent radiotherapy.

Unfortunately, one patient succumbed to metastatic disease during follow‐up, unrelated to his radical orchidectomy and *ex vivo* onco‐microTESE. At the time of reporting, all other patients for whom follow‐up data were available are in remission.

### Overall Sperm Retrieval Rates

Onco‐microTESE of the affected testis successfully surgically retrieved sperm in 25/42 testes (60%) and in 25/38 patients (66%) across this entire series. Sperm was successfully surgically retrieved in a further two of nine patients from the contralateral testis, giving a final successful fertility preservation rate via SSR of 27/38 patients (71%). Considering those who had additional successful fertility preservation after hormone stimulation and no further surgery, the total fertility preservation rate in this entire consecutive series was 28/38 patients (74%). To date none of the patients had attempted to utilise their stored sperm for ART.

### Group 1: Patients Presenting with Azoospermia

Onco‐microTESE was successful at retrieving sperm in 19/35 testes (54%), and 19/31 patients (61%) presenting with azoospermia. All four patients with azoospermia with bilateral synchronous tumours had successful SSR by onco‐microTESE. Seven of 10 patients with azoospermia and tumour in a solitary functioning testis had a successful SSR by onco‐microTESE.

Of the 12 patients in this sub‐group with failed onco‐microTESE, eight had a contralateral microTESE of the unaffected testis in the same operation; sperm was successfully retrieved in a further two of eight cases. Three of the patients that had failed onco‐microTESEs declined contralateral microTESE.

One of the six patients who did not have a contralateral microTESE at the same time was not offered the procedure as he was hypogonadic at the time of his onco‐microTESE (pre‐orchidectomy FSH 29iU/L, LH 15iU/L, testosterone 4.1 nmol/L). Clomiphene citrate therapy was commenced after onco‐microTESE (50 mg alternate days) and after 3 months he had an increase in his testosterone to 16.6 nmol/L. By 6 months his semen analysis showed occasional spermatozoa in his ejaculate, and after 9 months of clomiphene citrate he achieved total sperm counts of 2.8 million/mL in his ejaculated samples, which was successfully used with ICSI resulting in a live birth [21].

The overall total successful fertility preservation rate in all patients with azoospermia (by ipsilateral onco‐microTESE, contralateral microTESE or collection of ejaculated sperm after stimulation of the remaining testis with clomiphene citrate) was therefore 22/31 (71%). The range of the number of vials retrieved in the azoospermic cohort was 1–6, with a median (IQR) of 2 (2–3.25) vials.

The preoperative biochemical variables (AFP, beta‐hCG, LDH, total testosterone, FSH or LH) between those that underwent successful vs unsuccessful SSR are presented in Table 2. A total of five patients were missing data in one or more of these key variables.

**Table 2:** Comparison of variables in patients with azoospermia who underwent successful and unsuccessful SSR.

Variable	Successful SSR (*N* = 19)	Unsuccessful SSR (*N* = 16)	No. of missing data points
Age, years, mean (sd)	32.1 (5.4)	33.5 (4.2)	
AFP (normal range: 0–5.8 kU/mL), kU/mL, median (IQR)	3.0 (1.7–3.8)	3.3 (1.9–8.3)	3
Beta‐hCG (normal range: 0–2 mIU/mL), mIU/mL, median (IQR)	<1.0 (0–2.5)	<1.0 (0–1)	1
LDH (normal range: 140–225 U/L), U/L, median (IQR)	185 (161–203)	207 (157–242)	2
Total testosterone (normal range: 9.9–30 nmol/L), nmol/L, mean (sd)	11.6 (5.4)	11.0 (5.7)	1
FSH (normal range: 1–12.4 IU/L), IU/L, median (IQR)	25.2 (9.6–32.0)	26.0 (20.7–33.1)	1
LH (normal range: 1–8.6 IU/L), IU/L, median (IQR)	10.0 (4.0–16.3)	11.1 (8.7–16.3)	1
Testes volume on US, mL, median (IQR)	6.8 (4.9–10.1)	9.4 (7.0–17.5)	
Tumour volume on US, mL, median (IQR)	0.9 (0.4–2.0)	2.2 (1.4–6.3)	
PTTO on US, %, median (IQR)	14.0 (8.0–33.5)	29.0 (21.5–41.5)	
Maximum tumour length on US, cm, median (IQR)	1.2 (1.1–1.8)	2.0 (1.4–2.5)	
Time from presentation to operation, days, mean (sd)	9.5 (1.5)	11.2 (1.7)	

The median (IQR) tumour volume on US in the successful SSR group was more than half the tumour volume in the unsuccessful SSR group 0.9 (0.4–2.0) mL compared to 2.2 (1.4–6.3) mL. Likewise, the median (IQR) PTTO on US was 14% (8.0–33.5%) and 29% (21.5–41.5%) for successful SSR and unsuccessful SSR, respectively.

### Group 2: Patients Presenting with Severe OAT

Onco‐microTESE was successful at securing further sperm in six of seven testes and six of seven patients with severe OAT. In the one case of failed onco‐microTESE, a contralateral microTESE was performed on the remaining smaller atrophic testis, which was also unfortunately unsuccessful. The overall range of number of vials retrieved in the severe OAT cohort was 1–4, with a median (IQR) of 2 (1–3) vials.

## Discussion

### Overall Success Rates

This study has shown that onco‐microTESE is an excellent option for patients presenting with suspected TC who are azoospermic or have very severe OAT. Sperm can be retrieved from the affected testicle with high rates of success (66% in our overall cohort). An additional contralateral microTESE of the unaffected testicle, or hormone stimulation of the remaining hypogonadal testis, resulted is a successful sperm retrieval in a further three cases, leading to final successful fertility preservation in 74% of all cases [20]. To our knowledge these data represent the largest cohorts of patients undergoing onco‐microTESE and one of the highest success rates across the published literature. The overall success rate of initial onco‐microTESE was comparable to those seen in microTESE performed for azoospermia in the non‐cancer setting [21].

**Fig. 1:**
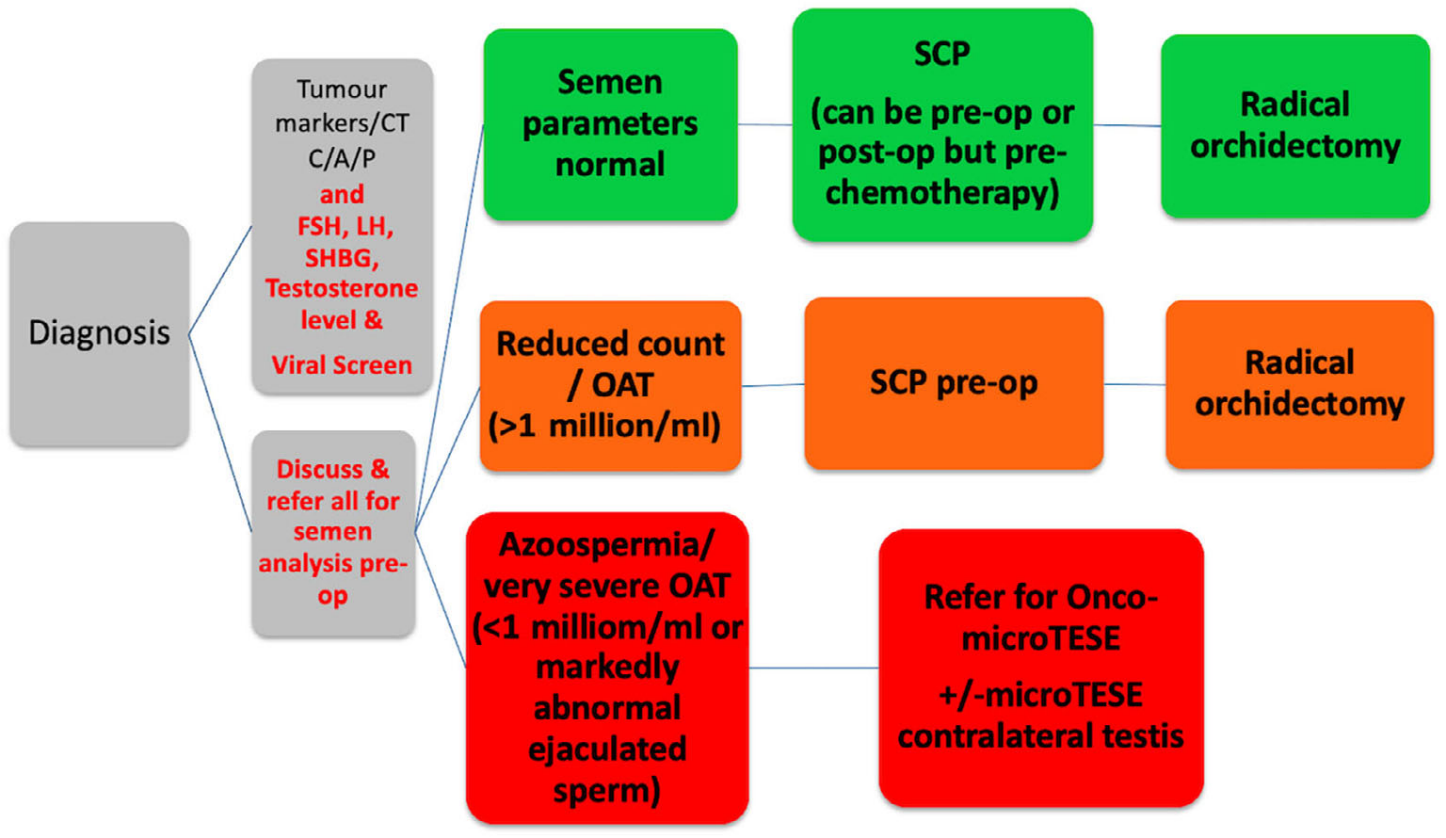
Recommendations for a new treatment paradigm for patients with suspected TC and sub‐fertility (from Shabbir and Fanshawe [23]). post‐op, postoperatively; pre‐op, preoperatively; SCP, semen cryopreservation; SHBG, sex hormone‐binding globulin.

Onco‐microTESE combines fertility preservation with oncological management; however, it relies on early assessment of patient's testicular function and semen analysis to identify those who would benefit from this approach. Whilst it is standard practice for patients with suspected TC to have assessment of tumour markers at first presentation, assessment of testis function markers, namely hormone profile and semen analysis, are not typically considered essential before surgery [22]. Knowledge of the baseline function allows a more patient‐centred approach to managing the impact of treatment on the development of hypogonadism and sub‐fertility and allows alternative options for maximising fertility potential. Figure 1 [23] shows a proposed management paradigm for patients with suspected TC and impaired fertility.

Studies have reported that only approximately 50–60% of oncologists and urologists discussed fertility preservation options with their patients, with common reasons for the omission being a lack of awareness of available options, uncertainty regarding access to a fertility preservation service, and insufficient time to achieve fertility preservation before treatment [6, 24, 25]. Uptake of fertility preservation is increased considerably when recommended by a healthcare professional [6]. Education of clinicians on this topic is crucial to allow the early identification of sub‐fertile patients with TC and ensure they are encouraged to explore fertility preservation options. In our series, overall fertility preservation in the most difficult sub‐group of patients presenting with azoospermia (by means of either onco‐microTESE, contralateral micro‐TESE or collection of sperm in a patient previously azoospermic following clomiphene citrate therapy) was 22/31 patients (71%). In our study, the median time from the point of referral or presentation to surgery was 7 days, emphasising that even patients with azoospermia can achieve a high rate of fertility preservation in a timely manner without significantly delaying oncological management, in keeping with the findings of previous studies [23]. Previous studies have identified a lack of evidence to suggest that delaying surgery for fertility preservation results impacts oncological outcomes [26, 27].

Comparing our cohort of patients with other case reports and series on oncoTESE represents a challenge. This is due to the heterogeneity of what the term ‘oncoTESE’ has been used to describe in the literature over the years, ranging from a conventional or microTESE from the contralateral unaffected testes at the time of orchidectomy, to an ipsilateral conventional or microTESE from the affected testicle at the time of radical orchidectomy. In all, 45 such ‘oncoTESE’ cases have been reported across 16 small case series and case reports, with an overall successful SSR rate of 69% [28, 29, 30, 31, 32, 33, 34, 35, 36].

Kaul et al. [37], reported a series of 26 patients with TC and azoospermia who underwent ipsilateral onco‐microTESE with a success rate of only 35% (nine of 26). Of the unsuccessful patients, a further three underwent successful contralateral microTESE, giving a final fertility preservation rate of 46% (12 of 26). It is not clear why the onco‐microTESE sperm retrieval rate in this study was lower than that achieved in our present series. The ipsilateral success rate was also notably lower than previous studies, which reported areas of mature spermatogenesis in 70% of testes affected by TC [38]. However, the series did report on three patients who utilised their retrieved sperm with assisted reproduction to achieve two successful live births, although it is not clear if this was achieved by using ipsilateral or contralateral retrieved sperm.

Blecher et al. [38], reported a case series of 13 patients presenting with testicular masses of which 12 were azoospermic and one had severe OAT (<1 million sperm/mL). They utilised the same onco‐microTESE technique as in our present series; however, it is not clear if any contralateral microTESE was delayed or contemporaneous. Successful SSR was reported in seven of 13 onco‐microTESE, with a further five of eight having a successful contralateral microTESE, giving an overall fertility preservation success rate of 10 of 13 patients. These results are in keeping with the results of our present series. The series also reported six pregnancies with five live births. However, once again it is not clear if this was from onco‐microTESE or contralateral microTESE.

Typically, if spermatozoa of sufficient quality and quantity were identified in the ipsilateral testis, contralateral microTESE was not performed. Whilst it could be argued that microTESE could be undertaken at the time of contralateral biopsy to assess for germ cell neoplasia *in situ* (GCNIS; using the accepted criteria for contralateral biopsy in TC as defined in the EAU guidelines [20]), the risks of complication for an extended sperm search would be slightly higher than if a standard testis biopsy looking for GCNIS was performed alone. This would not be for any additional benefit when adequate sperm had already been retrieved. Nonetheless, in patients that may require future chemotherapy with its associated gonadotoxic effects, thorough counselling regarding the risks and benefits of a contralateral microTESE should be discussed with patients on an individual basis.

Moody et al. [39] found spermatogenesis was present in 70% of testes removed for TC. However, spermatogenesis was only found in focal areas in 38% of cases. If these patients underwent random peripheral sampling using a standard TESE approach, these focal areas may be missed resulting in suboptimal fertility preservation and increased need for a contralateral procedure. Although TESE is more widely available than microTESE, the latter remains the EAU recommended approach to securing sperm [20], and we would only advocate microTESE for SSR to optimise fertility preservation. Development of specialist regional centres of fertility preservation excellence who can perform onco‐microTESE for the selected number of patients who require this surgery is entirely feasible, as has similarly been achieved for microTESE.

### Predictors of Success

Moody et al. [39] reported that the presence of active spermatogenesis is inversely correlated with the percentage of tumour invasion found on histopathological specimens. Furthermore, Suzuki et al. [18] demonstrated testicular tumour diameter is negatively correlated with overall spermatogenesis, and spermatogenesis is more likely to be preserved in seminiferous tubules that are increasingly distant from the tumour. Choy et al. [40] observed spermatogenesis in 86%, 81%, and 57% of cases involving tumours of 1, 2, and 5 cm, respectively. Our findings whereby median tumour volume on US was 0.9 mL in the successful group compared with 2.2 mL in the unsuccessful group provides clinical support to previous histopathological findings. Further studies with greater patient numbers are required to elucidate this correlation. However, we present three cases of successful SSR when tumour volume exceeded 7.5 mL indicating that patients should still be considered for onco‐microTESE even in the context of large tumours.

Although onco‐microTESE was attempted in all patients in our cohort, in a situation with no preserved testicular parenchyma due to replacement with a very large tumour, ipsilateral onco‐microTESE would not be feasible. This emphasises the importance of consenting patients for the possibility of a contralateral microTESE at the time of their onco‐microTESE surgery.

Our results found no differences for tumour or hormonal markers between patients with TC and azoospermia that underwent successful or unsuccessful SSR. The results reported by Kaul et al. [37] and Blecher et al. [38] similarly found no differences or clear predictors of success. Furthermore, no role for raised tumour markers in predicting decreased spermatogenesis has been found in histopathology studies of TC specimens [39, 40]. Interestingly, the findings of significantly elevated FSH across the total cohort may potentially suggest the azoospermia may have predominantly developed as a result of progressive testicular failure as opposed to direct toxicity from the tumour itself.

### Limitations

The authors acknowledge that there is a lack of data on the utilisation of sperm for ART, in part due to the retrospective nature of the review with respect to the final fertility outcomes after sperm retrieval and in part due to the return of some patients to their local referral centres for ongoing follow‐up and fertility treatments. Due to the expedience with which preoperative investigations are undertaken for patients presenting with TC, the timing of hormone blood tests is not consistently early morning and patients were not starved, which may result in some variability of the hormone levels recorded. Similarly, genetic testing was not undertaken in this patient cohort as results typically take up to 8 weeks in our setting to return a result. This would result in an unacceptable delay in treating a patient with a testicular mass suspicious for TC and would not alter the decision to proceed with onco‐microTESE.

### Unifying Definition

The authors call for a uniform definition of onco‐microTESE in the context of TC. In the evolving literature the technique utilised in this study appears to show greater rates of SSR vs the alternatives [37, 38]. To allow for meta‐analysis between case reports and series a strict definition of surgical technique and outcome measure of success is required to further our understanding and knowledge of predicting factors in this field of onco‐fertility. The technique utilised in the present series avoids the need to operate on the contralateral testis except when no sperm are found during ipsilateral onco‐microTESE, therefore minimising overall testicular damage while combining fertility preservation and oncological treatment into one operation.

## Conclusion

This study has shown that men presenting with suspected TC and azoospermia can still achieve fertility preservation without significantly delaying their initial orchidectomy. In all, 61% of men with azoospermia achieved fertility preservation by onco‐microTESE alone. When combined with a contralateral microTESE, or hormone stimulation of a hypogonadal contralateral testes, the rate of successful fertility preservation increased to 71% in men with azoospermia. In men with severe OAT, an onco‐microTESE allowed additional fertility preservation from the testis being removed in six of seven of cases.

This study supports the need for routine integration of testicular function assessment and semen analysis into diagnostic pathways before intervention to identify those with sub‐fertility who may benefit from onco‐microTESE.

## Disclosure of Interests

None declared.
